# Hemostatic Markers in Congestive Heart Failure Dogs with Mitral Valve Disease

**DOI:** 10.1155/2014/589873

**Published:** 2014-10-16

**Authors:** Kreangsak Prihirunkit, Amornrate Sastravaha, Chalermpol Lekcharoensuk, Phongsak Chanloinapha

**Affiliations:** ^1^Department of Pathology, Faculty of Veterinary Medicine, Kasetsart University, Bangkok 10900, Thailand; ^2^Department of Small Animal Sciences, Faculty of Veterinary Medicine, Kasetsart University, Bangkok 10900, Thailand; ^3^Veterinary Teaching Hospital, Kasetsart University, Bangkok 10900, Thailand

## Abstract

Prothrombin time (PT), activated partial thromboplastin time (APTT), fibrinogen, D-dimer, antithrombin III (AT III), protein C (PC), factor VII (F.VII), and factor VIII (F.VIII), as well as hematocrit (HCT), platelets number (PLT), total plasma protein (TP), and albumin (ALB), were studied on fifty-eight congestive heart failure (CHF) dogs with mitral valve disease (MVD) and fifty control dogs. All of variables of MVD group, except APTT, were significantly different (*P* < 0.5) from control group. The variables were also compared among functional classes of CHF dogs and control dogs. It was determined that the higher the functional class of CHF dogs was, the greater the levels of fibrinogen and D-dimer were, whereas the lesser the activities of AT III and PC were presented. Additionally, TP had linear correlation with fibrinogen, D-dimer, HCT, and PLT (*r* = 0.31, 0.30, 0.43, and 0.38, resp., *P* < 0.5). These findings suggested that fibrinogen and D-dimer were the factors predisposing hypercoagulability through an increase in blood viscosity. The hemorheological abnormalities would shift an overall hemostatic balance toward a more thrombotic state in CHF dogs with MVD.

## 1. Introduction

Degenerative valve disease is one of the most common forms of canine heart disease and is also known as endocardiosis [1]. The most commonly affected valve in the dog is the mitral valve [2]. It is a well-compensated disease with a long evolution [3]. A genetic tendency to develop the disease has been proved in Cavalier King Charles Spaniel and Dachshunds [4]. Grossly, mitral valve disease (MVD) is a markedly thickened valve with the swollen free edge. As the disease progresses, the regurgitation of blood from the ventricle into the atrium causes volume overload and possibly leads to congestive heart failure (CHF) [5].

CHF has been associated with the profound clinical effects of hemostasis. The high plasma markers of thrombin activity, fibrinolytic activity, and platelet activation have been reported [6, 7]. Even though heart failure in human is associated with thromboembolic stroke risk, the prevalence of overt clinical thromboembolism in dogs with CHF is rarely reported [8].

Thromboembolic complications have been attributed to an imbalance between procoagulant and anticoagulant factors, including thrombocytosis, hemoconcentration, hyperviscosity, and immobilization [9]. Protein C (PC) and antithrombin III (AT III) are major natural anticoagulants. They play an important role in preventing excessive coagulation, whereas fibrinogen and D-dimer indicate the thromboembolic tendency [10].

The diagnosis of hypercoagulation is essential for identification of individuals at higher risk of thrombosis and for an early treatment of thrombotic disorders. Since the investigations relevant to the determination of coagulation in dogs with MVD remain scanty, the aims of the present study therefore are (1) to compare the levels of hemostatic markers between the MVD and the control dogs and (2) to investigate the alteration of coagulation parameters among groups of functional class of CHF dogs.

## 2. Materials and Methods

### 2.1. The Study Site

The study was carried out at the Veterinary Teaching Hospital, Kasetsart University, Thailand. In total, 108 dogs were categorized into MVD group (*n* = 58) and control group (*n* = 50). On the basis of physical examination, chest radiography, electrocardiography, and echocardiographic performance with cardiac alteration score, as previously described [5], 58 CHF dogs with MVD were selected from cardiology clinic. All patients were diagnosed and classified as the functional class of CHF by only one cardiologist. To classify CHF in dogs, New York Heart Association (NYHA) Classification was applied [11]. Patients were excluded if they had other thrombotic risk factors, such as infection, disseminated intravascular coagulation, diabetes, Cushing's disease, and neoplasm. Complete blood count (CBC) was performed to exclude ongoing inflammation. Dogs in control group were recruited from blood donors and were checked up by veterinarians as clinically healthy. None of the dogs received antithrombotic agents within 6 months prior to sample collection.

### 2.2. Collection and Preparation of Blood Samples

In each patient and each control dog, a CBC was conducted from the blood collected under aseptic conditions in tubes containing K_3_EDTA as an anticoagulant. Clotted blood samples were used for clinical chemistry analysis, including total plasma protein (TP) and albumin (ALB). Blood samples for a coagulation profile: prothrombin time (PT), activated partial thromboplastin time (APTT), fibrinogen, D-dimer, antithrombin III (AT III), protein C (PC), factor VII (F.VII), and factor VIII (F.VIII), were collected using siliconized vacutainer tubes containing 3.18% trisodium citrate as an anticoagulant with a ratio of 9 : 1 (vol/vol). The samples were centrifuged at 4,500 rpm for 15 min to obtain platelet-poor plasma and were stored at −80°C until evaluation.

### 2.3. Sample Analysis

The CBCs were done by an automated hematology analyzer (Cell-Dyn 3500, Abbot Diagnostics, Illinois, USA); blood chemical tests were evaluated by an automated chemical analyzer (Liasys, AMS Diagnostics, Rome, Italy). According to the manufacturer's guidelines, the coagulation profiles were assayed, using Dade Behring (Marburg, Germany) diagnostic kits. The coagulation tests were carried out on an automated blood coagulation analyzer (Sysmex CA-1500, Kobe, Japan) with coagulometric, chromogenic, and immunoturbidimetric methods. PT and APTT were measured automatically as clotting tests using commercial reagents. The test results were reported in seconds. Fibrinogen concentration was determined by clotting assay. The enzyme thrombin converted the soluble plasma fibrinogen into its insoluble fibrin and was then inversely proportional to the fibrinogen concentration by a comparison with the fibrinogen calibration curve [12]. D-dimer concentration was measured with an immunoturbidimetric method. The assay detected an antigen-antibody reaction according to the endpoint method. The result was reported as *μ*g/mL fibrinogen equivalent units. Measurement of both F.VII and F.VIII was performed in PT and APTT assays with a mixture of the F.VII and F.VIII deficient substrate plasmas, respectively. The results were interpreted, using a standard calibration curve. AT III and PC activities were assessed by functional chromogenic assay. Two steps for AT III activity measurement were performed. First, the test plasma was incubated with an excessive amount of thrombin reagent in the presence of heparin. Second, the color was measured by the reaction between the chromogenic substrate and the residual thrombin after being neutralized by AT III in the sample. The color of the reaction was inversely proportional to the AT III level monitored kinetically at 405 nm. The percentage activity was read using the standard calibration curve [13]. For the measurement of PC, a specific snake venom activator was used to activate PC in the sample which was converted to activated PC. Then, the color of the reaction was proportional to the PC level monitored kinetically at 405 nm. The percentage activity was read by the standard calibration curve [14].

All of calibration curves prepared for the coagulation profile were modified using canine-pool plasma instead of commercial, lyophilized human plasma. Thirty canine plasma were mixed and used as normal pooled plasma. To generate the standard curves of AT III, PC, F.VII, and F.VIII, the canine-pool plasma was assigned as 100% activity. Subsequently, it was diluted through a dynamic linear range from 0% to 100% activity of each assay.

### 2.4. Statistical Analysis

Data were analyzed by the NCSS software [15]. The results of all variables: PT, APTT, fibrinogen, D-dimer, AT III, PC, F.VII, F.VIII, hematocrit (HCT), platelets number (PLT), TP, and ALB, were expressed as a mean ± SD. Differences of these variables between MVD and control groups and among functional classes of CHF dogs were determined by using a general linear model. Age, weight, and breed were not included in the model as covariates for multivariate analysis. If the model showed significance, Fisher's LSD multiple comparison was used to compare the means. Spearman's rank correlation coefficient (*r*) was used for the correlation analyses. The linear relationship was interpreted as follows: *r* between 0 and 0.3 (0 and −0.3) indicated a weak positive (negative), *r* between 0.3 and 0.7 (−0.3 and −0.7) indicated a moderate positive (negative), and *r* between 0.7 and 1.0 (−0.7 and −1.0) indicated a strong positive (negative) linear relationship, respectively. All tests were two-tailed, and a value with *P* < 0.05 was considered of statistical significance.

## 3. Results

Fifty-eight CHF dogs with MVD were included in final analyses. They were consisting of 38 males and 20 females of various breeds, including 1 Dachshund, 1 Spitz, 2 Miniature Pinschers, 2 Golden Retrievers, 3 Dalmatians, 14 Shih Tzus, 14 mixed breeds, and 21 Poodles. On the criterion of NYHA, the CHF dogs were classified into Class I (*n* = 2), Class II (*n* = 19), Class III (*n* = 32), and Class IV (*n* = 5). Besides, 50 clinically healthy dogs (33 males and 17 females) were enrolled in the control group. They consisted of 3 Siberian Huskies, 4 German Shepherds, 4 Labrador Retrievers, 14 Golden Retrievers, and 25 mixed breed dogs. Descriptive data is demonstrated in Table 1.

**Figure 1:**
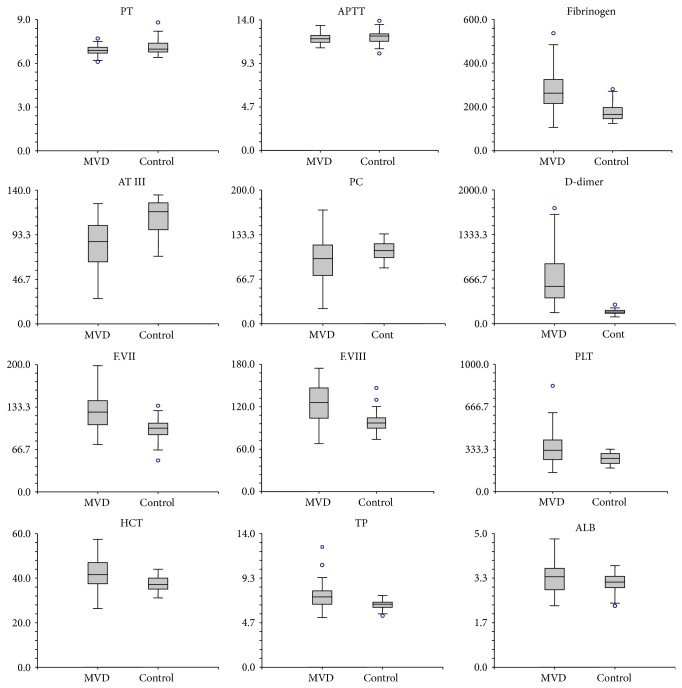
Comparison of all parameters; prothrombin time (PT), activated partial thromboplastin time (APTT), fibrinogen, antithrombin III (ATIII), protein C (PC), D-dimer, factor VII (F.VII), factor VIII (F.VIII), platelets number (PLT), hematocrit (HCT), total plasma protein (TP), and albumin (ALB), between dogs with mitral valve disease (MVD) and control (cont.) dogs observed in the study. Data are presented as boxes and whiskers. Each box includes the interquartile range, whereas the line within a box represents the median; the whiskers represent the range and extend to a maximum of 1.5 times the interquartile range. Outliers are depicted by circles.

The comparison of selected coagulation, hematology, and blood chemistry profiles: PT, APTT, fibrinogen, D-dimer, AT III, PC, F.VII, F.VIII, hematocrit (HCT), platelets number (PLT), TP, and ALB, between MVD and control groups showed that only APTT was not statistically different (*P* > 0.05) (Figure 1). Those variables were also compared among functional classes of CHF dogs and control dogs. They were summarized in Table 2. It was shown that the more the functional class of CHF dogs was, the more the concentration of fibrinogen and D-dimer increased, whereas the lesser the activity of AT III and PC decreased. However, these were noted significantly (*P* < 0.05) in Class IV CHF dogs. PT of CHF Class III and Class IV was statistically less than that of dogs in control group (*P* < 0.05), but APTT was not significantly different (*P* > 0.05) among functional classes of CHF group and control group. According to the percentage activities of F.VII and F.VIII, only Class I CHF dogs had no significant difference (*P* > 0.05) compared to that of control dogs, while the levels of HCT, PLT, TP, and ALB of CHF dogs in Class II and Class III were of significant difference (*P* < 0.05) compared to that of dogs in control group.

The simple correlation among variables of the 58 dogs with CHF caused by MVD is shown in Table 3. Fibrinogen correlated positively with D-dimer, F.VII, F.VIII, and TP (*r* = 0.54, 0.36, 0.37, and 0.31, resp.; *P* < 0.05), but negatively correlated with AT III and PC (*r* = −0.44 and −0.33, resp.; *P* < 0.05). D-dimer negatively correlated with AT III and PC (*r* = −0.57 and −0.36, resp.; *P* < 0.05), whereas it was positively correlated with F.VII, F.VIII, HCT, PLT, and TP (*r* = 0.44, 0.49, 0.33, 0.31, and 0.30, resp.; *P* < 0.05). Additionally, a linear correlation was found between hematologic and protein variables.

## 4. Discussion

Disturbance in hemostasis was a common complication of CHF. In human, the incidence of hypercoagulability was a surrogate for clinical events which potentially caused thromboembolism and mortality in heart failure patients [16]. The thromboembolic complication was not found in the current study, judged by the lack of clinical signs; however, the hypercoagulable parameters were observed. The hemostatic markers: fibrinogen, D-dimer, AT III, and PC, were significant difference between MVD and control groups. Prothrombotic parameters: fibrinogen and D-dimer, concentration increased, whereas natural anticoagulants: AT III and PC, decreased. We also found that the alteration of hemostatic variables related to the severity of heart failure. The higher the functional class of CHF group was, the greater the levels of fibrinogen and D-dimer were, whereas the lesser the activities of AT III and PC presented. Moreover, correlations among these parameters were remarked. PT and APTT were screening tests and did not correlate with other variables. Likewise, F.VII and F.VIII were not statistically different among NYHA classes of CHF group, but they trended to have an increased percentage activity in higher functional class of CHF dogs. There were also significant linear correlations with fibrinogen and D-dimer; therefore these two variables were supportive laboratory findings.

The altered hemostatic markers suggested that they were important in disease progression and thromboembolic complication. An increased fibrinogen concentration reflected quite well an increased coagulation potential. D-dimers were a specific degradation product of plasmin-cleaved cross-linked fibrin and, therefore, were considered a more specific indicator of hypercoagulable condition [17]. Even though the human D-dimer was applied in the current study, it was able to cross-react with canine D-dimer [18]. AT III and PC were major natural anticoagulants. A decreased level of AT III and PC enhanced thrombin generation which increased coagulation activity [19].

As a result of a further reduction in cardiac output and a worsening of abnormalities in regional blood flow, a hypercoagulable state in patients with CHF was associated with a reduction in plasma volumes [20]. The current study was undertaken to determine the hemorheology of the dogs with CHF. Blood viscosity was determined by an increased level of TP. It significantly increased and positively correlated with fibrinogen, D-dimer, HCT, and PLT. The result indicated that the hematological change has been along with an elevated marker of fibrin turnover: fibrinogen and D-dimer. Although it is important to realize that an increase in plasma fibrinogen concentration may occur as a result of transient fluid shift out of the intravascular space, leading to hemoconcentration, but D-dimer concentration was elevated only by the degradation of cross-linked fibrin [17]. A manifestation of hyperfibrinogenemia may be a factor predisposing hypercoagulability through increase in blood viscosity and formation of platelet aggregation [21]. Together with the viscosity, the increase in hematologic parameters would shift an overall hemostatic balance toward a more hypercoagulable state in the dogs with CHF [22].

Limitation of the current study was that the dogs with MVD and control dogs were not strictly matched regarding breed, age, and sex. Within the MVD group, dogs were small to medium breeds, while the control dogs were recruited from blood donors which were mainly medium to large breed dogs. Moreover, average age of MVD group was older than that of control dogs. Coincidentally, the studied population was male dominant. Based on physical examination and echocardiographic evaluation, a limited number of dogs with NYHA Class I and Class IV were made. Because of mild clinical signs with unlimited physical activity, CHF Class I dogs in this study were found incidentally. On the other hand, a complication was usually found in CHF Class IV dogs. Therefore, these dogs were excluded. Finally, it was the fact that an increased fibrin turnover may also enhance platelet aggregation. Anyhow, the platelet function test was not available in our settings.

## 5. Conclusion

To a lesser extent of the relationship between thromboembolism and MVD in veterinary study, the present study added up knowledge regarding the hemostatic markers in CHF dogs caused by MVD. The most important determinants of prothrombotic state were increased fibrinogen and D-dimer concentrations and decreased AT III and PC activities. Additionally, the abnormalities of hemorheological function would deteriorate hypercoagulability in CHF dogs with MVD.

## Figures and Tables

**Table 1:** Demographic data from 50 control dogs and 58 CHF dogs with MVD.

	Control (*n* = 50)	NYHA classification				Total CHF (*n* = 58)
		I (*n* = 2)	II (*n* = 19)	III (*n* = 32)	IV (*n* = 5)	
Age (year)	5.5 ± 1.4	13 ± 4.2	10.2 ± 3.4	10.8 ± 3.0	11.6 ± 2.1	10.8 ± 3.1
Weight (Kgs)	33.3 ± 8.4	17.1 ± 10.5	8.5 ± 6.4	7.3 ± 5.4	15.8 ± 10.5	8.8 ±6.8
Sex (male/female)	33/17	1/1	14/5	18/14	5/0	38/20

**Table 2:** Comparison of selected coagulation, hematology, and blood chemistry parameters among various NYHA functional classes of CHF group and control group.

	Control (*n* = 50)	NYHA functional class				Total patients (*n* = 58)
		I (*n* = 2)	II (*n* = 19)	III (*n* = 32)	IV (*n* = 5)	
PT (sec.)	7.1 ± 0.5^1a^	7.0 ± 0.1^abc^	6.9 ± 0.3^ab^	6.8 ± 0.3^bc^	6.5 ± 0.3^c^	6.9 ± 0.3^2^
APTT (sec.)	12.2 ± 0.7^1a^	12.3 ± 0.3^a^	12.0 ± 0.4^a^	12.0 ± 0.6^a^	11.7 ± 0.4^a^	12.0 ± 0.5^1^
Fibrinogen (mg/dL)	175.2 ± 37.4^1a^	223.5 ± 55.0^ab^	268.8 ± 88.5^b^	275.9 ± 99.5^b^	351.8 ± 83.9^c^	278.3 ± 94.9^2^
D-dimer (*μ*g/mL)	117.2 ± 36.9^1a^	197.0 ± 45.3^ab^	419.2 ± 173.2^b^	742.8 ± 300.6^c^	1349.8 ± 445.4^d^	670.3 ± 380.7^2^
ATIII (%)	112.6 ± 16.5^1a^	88.7 ± 4.5^abc^	87.2 ± 21.0^b^	84.7 ± 27.1^b^	59.2 ± 23.1^c^	83.5 ± 25.2^2^
PC (%)	110.2 ± 12.9^1a^	99.8 ± 8.3^ab^	101.2 ± 24.6^ab^	93.3 ± 33.3^b^	57.3 ± 21.7^c^	93.0 ± 31.1^2^
F.VII (%)	99.9 ± 15.8^1a^	101.0 ± 8.5^ab^	124.6 ± 36.5^b^	125.8 ± 23.5^b^	132.9 ± 15.9^b^	125.2 ± 27.7^2^
F.VIII (%)	98.1 ± 12.9^1a^	112.4 ± 10.5^ab^	124.7 ± 29.0^b^	124.0 ± 26.0^b^	139.3 ± 10.2^b^	125.1 ± 25.8^2^
HCT (%)	37.4 ± 3.0^1a^	42.5 ± 2.3^ab^	42.4 ± 6.7^b^	41.8 ± 7.0^b^	41.0 ± 8.2^ab^	42.0 ± 6.7^2^
PLT (×10^3^/*μ*L)	259.8 ± 45.5^1a^	257.5 ± 109.6^ab^	359.5 ± 122.8^b^	355.5 ± 136.3^b^	304.8 ± 104.7^ab^	349.1 ± 128.0^2^
TP (g/dL)	6.5 ± 0.5^1a^	7.0 ± 0.3^ab^	7.6 ± 1.0^b^	7.5 ± 1.5^b^	6.7 ± 1.1^ab^	7.5 ± 1.3^2^
ALB (g/dL)	3.1 ± 0.3^1a^	3.4 ± 0.6^ab^	3.5 ± 0.6^b^	3.4 ± 0.5^b^	2.9 ± 0.5^a^	3.4 ± 0.6^2^

^1,2^Different superscript numbers indicate statistical significance between MVD group and control group at *P* < 0.05. ^a,b,c,d^Different superscript letters indicate statistical significance among various NYHA classes of CHF group and control group at *P* < 0.05.

**Table 3:** Correlation between variables of 58 CHF dogs with MVD.

	PT	APTT	Fibrinogen	D-dimer	ATIII	PC	F.VII	F.VIII	HCT	PLT	TP	ALB
PT	1.00											
APTT	0.12	1.00										
Fibrinogen	−0.09	−0.01	1.00									
D-dimer	−0.25	−0.24	0.54^a^	1.00								
AT III	0.22	0.13	−0.44^a^	−0.57^a^	1.00							
PC	0.09	0.01	−0.33^a^	−0.36^a^	0.24	1.00						
F.VII	−0.28	−0.08	0.36^a^	0.44^a^	−0.22	−0.19	1.00					
F.VIII	−0.26	−0.19	0.37^a^	0.49^a^	−0.25	−0.05	0.38^a^	1.00				
HCT	−0.04	0.07	0.20	0.33^a^	−0.26	−0.13	0.24	0.24	1.00			
PLT	−0.11	0.07	0.23	0.31^a^	−0.18	−0.15	0.17	0.18	0.34^a^	1.00		
TP	0.03	−0.20	0.31^a^	0.30^a^	−0.26	−0.12	0.14	0.19	0.43^a^	0.38^a^	1.00	
ALB	0.08	−0.01	0.06	0.14	−0.21	0.04	0.07	0.00	0.44^a^	0.43^a^	0.56^a^	1.00

^a^Significant difference at *P* < 0.05 and *r* between 0.3 and 0.7 (−0.3 and −0.7) indicate a moderate positive (negative) linear relationship.
